# Alzheimer’s disease progression characterized by alterations in the molecular profiles and biogenesis of brain extracellular vesicles

**DOI:** 10.1186/s13195-020-00623-4

**Published:** 2020-05-08

**Authors:** Xavier Gallart-Palau, Xue Guo, Aida Serra, Siu Kwan Sze

**Affiliations:** 1 Division of Chemical Biology & BioTechnology, School of Biological Sciences, Nanyang Technological University, 60 Nanyang Drive, Singapore, 637551 Singapore; 2 IMDEA-Food Research Institute, +Pec Proteomics, Campus of International Excellence UAM+CSIC, Old Cantoblanco Hospital, 8 Crta. Canto Blanco, 28049 Madrid, Spain; 3 Institut Investigació Sanitària Pere Virgili (IISPV), Hospital Universitari Institut Pere Mata, Reus, Tarragona Spain

**Keywords:** Alzheimer’s disease, Vascular dementia, Extracellular vesicles, Braak staging, Neurodegeneration, Autophagy, Neuroinflammation

## Abstract

**Background:**

The contributions of brain intercellular communication mechanisms, specifically extracellular vesicles (EV), to the progression of Alzheimer’s disease (AD) remain poorly understood.

**Methods:**

Here, we investigated the role(s) of brain EV in the progressive course of AD through unbiased proteome-wide analyses of temporal lobe-derived EV and proteome-label quantitation of complementary remaining brain portions. Furthermore, relevant proteins identified were further screened by multiple reaction monitoring.

**Results:**

Our data indicate that EV biogenesis was altered during preclinical AD with the genesis of a specific population of EV containing MHC class-type markers. The significant presence of the prion protein PrP was also manifested in these brain vesicles during preclinical AD. Similarly, sequestration of amyloid protein APP in brain EV coincided with the observed PrP patterns. In contrast, active incorporation of the mitophagy protein GABARAP in these brain vesicles was disrupted as AD progressed. Likewise, disrupted incorporation of LAMP1 in brain EV was evident from the initial manifestation of AD clinical symptoms, although the levels of the protein remained significantly upregulated in the temporal lobe of diseased brains.

**Conclusions:**

Our findings indicate that impaired autophagy in preclinical AD coincides with the appearance of proinflammatory and neuropathological features in brain extracellular vesicles, facts that moderately remain throughout the entire AD progression. Thus, these data highlight the significance of brain EV in the establishment of AD neuropathology and represent a further leap toward therapeutic interventions with these vesicles in human dementias.

## Introduction

Alzheimer’s disease (AD) is the largest global contributor to cognitive decline and dementia [1]. AD-diseased brains tend to accumulate senile plaques and neurofibrillary tangles in the temporal and neocortical regions, which are mainly formed by fibrillar amyloid-β (Aβ peptide) and hyperphosphorylated Tau, respectively [2]. Although these can be considered the most evident neuropathological signs of the disease and are widely used to define the course of illness [3–5], recent findings indicate that subtler molecular mechanisms might be implicated in the progression of AD [6] and that their deciphering can notably help to guide the development of viable therapeutics for this fatal disease.

AD progresses with a high degree of molecular complexity. Postmortem brains affected by AD commonly present neuropathological mixed comorbidities, including vascular alterations [7], amyloidosis, Lewy bodies, and synucleinopathy (see [8] for an extensive review). Furthermore, cognitively preserved aged brains often show a significant burden of senile plaques and other AD hallmarks [9]. Therefore, the identification of subtle specific molecular alterations that can contribute to AD spreading inevitably drives our focus toward the scrutiny of abnormal brain intercellular communication [6, 10–12].

Beyond the well-known occurring synaptic synergies and brain cell secretome, all cells in the central nervous system (CNS) can release and take up a wide array of tiny double-membraned structures (20 to 2000 nm) with membrane-associated interaction domains and packed with diverse molecular cargoes and cellular organelles [13, 14]. These vesicles are known as extracellular vesicles (EV), and they conform to a very dynamic and adaptive intercellular communication mechanism [13]. Additionally, EV may assist the cells in extracellularly removing unwanted material [13]. Although the study of EV in the CNS is still in its infancy [6], changes in EV biogenesis that can relate to AD progression have been conjectured [15], and protein-specific alterations in EV potentially related to the spreading of AD proteinopathy and neurotoxicity have been described [16]. Furthermore, EV may serve therapeutic purposes in AD [12, 17], and given their circulating nature, these vesicles can display biological marker functions [17]. However, to the best of our knowledge, proteome-wide investigation of alterations in brain EV composition and biogenesis related to AD progression is still pending. Thus, based on our experience with the application of discovery-driven proteomics approaches for the study of neurodegeneration [17–23] and state-of-the-art methodologies for the study of CNS-EV in vivo [14, 24], in this work, we investigate how AD progression could be underpinned by proteome-wide alterations in brain EV and by changes in the biogenesis of these vesicles.

## Methods

### Reagents

All reagents were purchased from Sigma-Aldrich (St. Louis, MO, USA) unless otherwise stated. Water and acetonitrile (ACN) of high-performance liquid chromatography (HPLC) grade were purchased from Thermo Fisher Scientific (Thermo Fisher Chemical, USA). Sequencing grade modified trypsin was obtained from Promega (Madison, WI, USA).

### Post-mortem brain tissues

Post-mortem brain tissues (Brodmann area BA21) were obtained from donors with histological signs at time of autopsy of AD, mixed dementias (Dem), or age-matched controls (control). All postmortem brain samples (*n* = 18) were generously provided by the Harvard Brain Tissue Resource Center (HBTRC, Boston, MA, USA). Histologically, AD cases displayed neurofibrillary tangles burden consistent with Braak stage III to VI (AD3 to AD6), whereas cases of mixed dementias displayed significant cerebrovascular lesions including small vessel disease predominantly affecting the temporo-parietal region. Clinical details of the donors, including age, post-mortem delay, and gender are provided in Supplementary Table 1. Brain tissues were frozen in liquid nitrogen at the time of autopsy and stored at − 150 °C until use. BA21 tissues were dissected in small pieces and large blood vessels were removed. Tissues were then washed three times with 1X PBS for 30 min. Three biological replicates were included per condition and were analyzed independently unless otherwise stated. Informed consent was obtained from all subjects or their legal representative. The use of post-mortem brain tissues was performed in strictly accordance with the declaration of Helsinki and the Singapore’s Human Biomedical Research Act (HBRA, Human Tissue Framework). Substantial manipulation of human tissues (as considered under Singapore’s HBRA 2019) was not performed in this study. All experimental procedures were performed in accordance to institutional guidelines.

### Processing of brain tissues prior to obtention of EV fractions

Brain tissues from each subject (~ 80 mg) were homogenized with detergent-free homogenization buffer (100 mM ammonium acetate (AA) supplemented with protease inhibitor cocktail tablets) preserving EV fractions [14, 25] and using the tissue homogenizer bullet blender (Next Advance, NY, USA) as previously described [14]. Next Advance metallic beads (0.9–2.0 mm of diameter) were washed per triplicate with 1X PBS during 30 min prior to mixing them with brain tissues at 1:1 ratio (w/w). All the detailed procedures for the obtention of EV brain fractions were performed at 4 °C. Brain homogenization was performed in four cycles with 300 μL of homogenization buffer and 5 min of homogenization per cycle. The first two cycles were conducted at medium intensity whereas the last two cycles were conducted at maximum intensity. At the end of each cycle, the homogenate was centrifuged at 15000×*g* for 10 min and the obtained supernatants were combined.

### Enrichment of brain EV by PROSPR

Brain EV were enriched from the detergent-free homogenates using PROSPR as previously described [14, 17]. Briefly, brain homogenates processed as previously indicated to minimize tissue contaminants in brain EV preparations [14] were mixed with four volumes of chilled acetone (− 20 °C), vortexed, and centrifuged at 5000×*g* for < 1 min. Supernatants containing hydrophobic EV fractions were then concentrated to near dryness in the vacuum concentrator (Concentrator Plus, Eppendorf AG, Hamburg, Germany).

### Label-free in solution digestion of brain EV fractions

PROSPR-isolated EV were dissolved in 16 M urea, 100 mM ammonium bicarbonate (ABB) buffer, then diluted twofold with HPLC water. EV constituent proteins were then digested as previously described with minor modifications [14]. Briefly, lysed EV were reduced with 20 mM dithiothreitol (DTT) at 30 °C for 3 h, followed by alkylation with 55 mM iodoacetamide (IAA) for 1 h at room temperature in dark environment. Samples were then diluted to < 1 M urea with 50 mM ABB. Overnight trypsin digestion was performed at 37 °C using a 1:20 enzyme-to-protein ratio (w/w) with sequencing grade modified trypsin. Proteolytic digestion was quenched by addition of 0.5% formic acid (FA). Tryptic digested peptides from EV fractions were then desalted using C18 Sep-Pak cartridge (Waters, Milford, MA). Elution of brain EV peptides was performed using 1 ml of 75% ACN, 0.1% FA. Eluates were then dried in the vacuum concentrator and reconstituted in 200 μL of 0.02% ammonium hydroxide (mobile phase A) for the subsequent fractionation by HPLC.

### Proteome extraction of complementary brain tissues

Complementary brain tissue proteome fractions that remained after processing brain tissues as described in the section “Processing of brain tissues prior to obtention of EV fractions” were processed to obtain the remaining brain proteomes in this study. Thus, brain protein fractions without EV fractions were further homogenized using homogenization buffer with addition of 1% sodium deoxycholate (SDC), as described above. Homogenization was performed by 5 homogenization cycles at maximum intensity using the bullet blender homogenizer until no obvious pellet was observable. Extracted proteins were then subjected to acetone precipitation mixing brain tissue homogenates with chilled acetone for 3 h before centrifugation at 20000×*g* for 10 min. Supernatants were subsequently discarded, and precipitated proteins were airdried to remove excess of acetone.

### In-solution tryptic digestion and isobaric peptide labeling of complementary brain proteomes

Complementary brain proteins were solubilized in lysis buffer pH 8.5 containing 1% SDC and 100 mM triethylammonium bicarbonate (TEAB) and supplemented with complete protease inhibitor cocktail. For whole brain characterization, brain proteins from independent subjects (*n* = 18) were pooled to obtain 200 μg protein/condition as previously described [18, 25]. Protein quantification was performed by bicinchoninic acid assay (BCA). Whole brain proteins were reduced with 10 mM tris 2-carboxyethyl phosphine hydrochloride for 2 h at 55 °C and subsequently alkylated using 55 mM IAA for 1 h at room temperature protected from the light. After alkylation, the SDC concentration was decreased to 0.5% using 100 mM TEAB. Overnight trypsin digestion was performed with sequencing-grade modified Promega trypsin at a 1:25 protein-to-enzyme ratio (w/w). Whole brain proteins digestion was subsequently quenched by addition of 0.5% final concentration of FA. SDC was removed from the digested samples by acidification, and peptides were further recovered from SDC precipitates as previously described [22, 26]. Tryptic peptides were dehydrated in vacuum concentrator and re-dissolved in 100 mM TEAB. Whole brain proteome peptides were labeled with 6-plex isobaric tags (tandem mass tag, TMT) from ThermoScientific (San Jose, CA) according to the manufacturer’s protocol. TMT isobaric group labels were distributed as follows: 126 age-matched controls (control), 127 mixed dementias (Dem), 128 preclinical AD (AD3), 129 early symptomatic AD (AD4), 130 advanced stage AD (AD5), and 131 final stage AD (AD6). Labeled peptides were subsequently desalted using a C-18 Sep-pack 200 mg cartridge (Waters, Milford, MA), and eluates were dried to dryness in the vacuum concentrator.

### High-pressure liquid chromatography fractionation

Brain EV and complementary brain proteomes were subjected to HPLC fractionation as previously described [21], but with a few modifications. Briefly, dried peptides were reconstituted in 200 μL of mobile phase A (10 mmol/L ammonium hydroxide in water) and fractionated using a Xbridge™ BEH130 C18 column (3.5 μm 4.6 × 250 mm, Waters, Elstree, UK) on a Shimadzu Prominence UFLC system (Dionex, Sunnyvale, CA) with UV monitoring of peptide intensities at 280 nm performed throughout. Peptide separation was performed at 1 mL/min using a 60-min gradient as follows: 0–5% B (0 mmol/L ammonium hydroxide in ACN) for 3 min, 5–35% B for 40 min, 35–70% B for 12 min, and 70–100% for 5 min. Fractions were collected at 1-min intervals and combined by concatenation. Concatenated fractions were then completely dried in the vacuum concentrator.

### Liquid chromatography tandem-mass spectrometry of brain EV proteomes

All experiments detailed were performed per triplicate generating three technical replicates for each biological sample analyzed. Dried peptide fractions of brain EV proteomes were reconstituted in mobile phase A (3% ACN, 0.1% FA) before label-free shotgun proteomics analysis by LC-MS/MS using a Dionex UltiMate 3000 UHPLC system coupled with an Orbitrap Elite mass spectrometer (Thermo Fisher, Inc., Bremen, Germany), as it was previously described with minor modifications [14, 21, 25, 27–29]. Spray was generated using an EASY-Spray ion source (Thermo Fisher Scientific, Inc.) working at 1.5 kV. Peptide separation was performed using a reverse-phase Acclaim PepMap RSL column (75 μm ID ×  15 cm, 2-μm particle size, Thermo Scientific, Inc.) maintained at 35 °C and working at 300 nL/min. The 60-min gradient used for peptide separation was as follows: 3% mobile phase B (90% ACN, 0.1% FA) for 1 min, 3 to 35% for 47 min, 35 to 50% for 4 min, 80% for 6 s, 80% (isocratic) for 78 s, 80 to 3% for 6 s, and then maintained at 3% for 6.5 min. Orbitrap Elite data acquisition was performed in positive mode using Xcalibur 2.2 software (Thermo Fisher Scientific, Inc., Bremen, Germany) alternating between full Fourier transform mass spectrometry (FT-MS; 350–2000 m/z, resolution 60,000, with 1 μscan per spectrum) and FT-MS/MS (150–2000 m/z, resolution 30,000, with 1 μscan per spectrum). Fragmentation of the 10 most intense precursors with charge > + 2 and isolated within a 2 Da window was performed using high-energy collisional dissociation mode using 32% normalized collision energy. A threshold of 500 counts was enabled. For full FT-MS and FT-MS/MS, automatic gain control was set to 1 × 10^6^.

### Liquid chromatography tandem-mass spectrometry of isobaric-labeled complementary brain proteomes

All experiments detailed were performed per triplicate generating three technical replicates for each biological sample analyzed. Dried sample fractions were carefully reconstituted in mobile phase A (3% ACN, 0.1% FA) before their analysis by LC-MS/MS using a Dionex Ultimate 3000 RSLCnano system coupled with a Q Exactive mass spectrometer (Thermo Fisher, Inc., Bremen, Germany). Spray was generated using an EASY-Spray ion source (Thermo Fisher Scientific, Inc.) working at 1.5 kV. Peptide separation was performed using a PepMap C18 column (3 μm, 100 Å, Thermo Fisher Scientific, Inc.) maintained at 35 °C and working at 300 nL/min. Separation of peptides was performed over a 60-min gradient with mobile phase A (0.1% FA in water) and mobile phase B (0.1% FA in 90% ACN) as follows: 3–30% B for 45 min, 30–50% B for 9 min, 50–60% B for 1 min, 60% B for 2 min, and finally maintained isocratic at 3% B for 3 min. Q Exactive data acquisition was perform in positive ion mode using Xcalibur 3.0.63 software (Thermo Fisher Scientific, Inc., Bremen, Germany) alternating full Fourier transform MS (FT-MS; 350–1600 m/z range, resolution of 70,000 at m/z 200, 1 μscan per spectrum) and FT-MS/MS (resolution 35,000) for the 10 most intense ions with charge > + 2 and isolated within a 2 Da window. Fragmentation of ions was performed by high-energy collisional dissociation fragmentation mode using 28% normalized collision energy. A threshold of 500 counts was enabled. For full FT-MS and FT-MS/MS, automatic gain control was set to 5 × 10^6^ and 2 × 10^5^, respectively.

### Targeted quantitation of selected proteins using liquid chromatography multiple reaction monitoring mass spectrometry

Identification of proteins in brain EV from individual subjects by label-free mass spectrometry proteomics was validated in individual subjects by liquid chromatography multiple reaction monitoring mass spectrometry (LC-MRM-MS). Multiple reaction monitoring (MRM) validation was performed using a TSQ Vantage triple quadrupole mass spectrometer coupled to an EASY-nLC 1000 nanoflow UHPLC system (Thermo Scientific Inc., Bremen, Germany). For every candidate protein, the 4–6 most intense transitions corresponding to *y*- or *b*-ions derived from fragmentation of the most high-confident peptide (precursor ion) were considered for protein validation. Precursor ions had an m/z < 1000 Da containing no cysteine residues and no missed cleaved sites, unless otherwise specified. Precursor ions were sorted based on their identification in brain EV from the label-free proteomics experiment performed using an Orbitrap Elite mass spectrometer. Transitions were also confirmed by in silico prediction using the Skyline software [30]. The transitions used in this study can be found in Supplementary Table 2. Protein relative quantification was based on average intensity of transitions obtained by MRM.

LC-MRM-MS experiment was performed per triplicate using specific in-solution digested and fractionated peptide mixtures. For each run, approximately 0.7 μg tryptic peptides were injected into an Acclaim PepMap RSLC C18 column (75 μm × 15 cm; nanoViper C18, 2 μm, 100 Å) (Thermo Scientific, USA) working at 35 °C. Mobile phase A (0.1% FA in HPLC water) and mobile phase B (0.1% FA in ACN) were used to establish a 60-min gradient with a flow rate of 300 nL/min as follows: initial conditions at 3% B, 3–30% at 45 min and increase to 50% from 45 to 54 min, increased to 60% in 3 min, and finally reverted to initial conditions at 3% B for re-equilibration.

Data acquisition was performed in positive ion mode. Ionization spray voltage was set at 1.1 kV in a Michrom Thermo CaptiveSpray nanoelectrospray ion source (Bruker-Michrom, Inc., Auburn, AL) with a capillary temperature of 250 °C and a discharge current of 4.0 μA. Quadrupole Q1 and Q3 peak width was set at 0.80 full width at half maximum. High-purity argon was used as the collision gas in Q2, and the collision energy (CE) for each MRM transition was optimized using Skyline software according to the equations: *0.030 × m/z + 2.905* (for 2+ charged peptides) or *0.038 × m/z + 2.281* (for 3+ charged peptides). Retention time scheduling was used for precursor detection based on the retention time detected in Orbitrap Elite.

### Western blot analysis of extracellular vesicle markers

Brain EV from subjects with AD obtained by PROSPR were subjected to western blotting of EV-specific markers by detection of the tetraspanin family marker CD9 and the exosomal-specific marker Alix. Three biological replicates were used, and western blot of brain EV preparations was performed as previously indicated with minor modifications [22]. Briefly, brain EVs were transferred into lysis buffer (1% SDS in H_2_O) and lysates were subjected to clearing centrifugation (16,000×*g*, 30 min) and protein quantification by BCA. Thirty micrograms of lysed protein from each subject were mixed in 4X Laemmli buffer; the mixture was boiled at 95 °C for 5 min and subsequently subjected to western blot analysis by resolving the proteins in 12% SDS-PAGE gel and blotting the resolved proteins onto nitrocellulose membranes. Cell Signaling Technology Inc. (MA, USA; Cat. # 2171; mouse monoclonal; 1:1000) and Santa Cruz Biotechnology (TX, USA; Cat. # sc-13118; mouse monoclonal; 1:500) were used as primary antibodies, while an appropriate HRP-conjugated anti-mouse secondary antibody from Jackson ImmunoResearch (PA, USA; Cat. # 115-035-003; 1:1000) was used as secondary antibody. Protein-antibody conjugates were then visualized by using the ECL chemiluminescence detection kit from Thermo Fisher Scientific (MA, USA).

### Characterization of extracellular vesicles by Nanoparticle Track Analysis

Vesicle content from brain EV enriched by PROSPR from subjects with AD (Braak AD3 *n* = 3) was subjected to Nanoparticle Track Analysis (NTA) as previously indicated [17]. Brain vesicle preparations were observed and analyzed through a Nanosight NS300 sCMOS instrument (Malvern, UK). Analysis parameters in Nanosight instrument were set as follows: capture time 60 s, camera level 4, slider shutter 50, slider gain 100, FPS 32.5, syringe pump speed 100, total volume per sample 1 mL, viscosity 0.906–0.910 cP, and temperature ~ 24 °C. No restricted areas were established during analyses; thus, all vesicle content in the sample was subjected to characterization.

### Bioinformatics and data analysis

Analysis of isobaric TMT labeling raw data files was performed using Proteome Discoverer 2.1 (Thermo Fisher, MA, USA) with Sequest HT and Mascot servers used in parallel during database search. User-defined parameters and false discovery rate (FDR) for assignation of peptides and proteins in Proteome Discoverer were set as previously specified, with minor modifications [18, 22]. Briefly, a FDR < 1% was set for searching using trypsin as proteolytic enzyme with a precursor mass tolerance of 10 ppm and a reporter ion integrated tolerance of 20 ppm. TMT 6 plex tag masses, identified according to the manufacturer’s description, were included as fixed modifications together with carbamidomethylation (C), oxidation (M), deamidation (NQ), trioxidation (CMWY), citrullination (R), and carbamylation (R). Phosphorylation (ST) and ubiquitination (K) were added as variable modifications. The UniProt human database downloaded on June 23, 2015, with 180,822 sequences and 71,773,890 residues was used for searching. A corrected ratio > 1.5-fold was considered to set significance for the upregulation threshold, and a corrected ratio < 0.5-fold was considered to set significance for the downregulation threshold in the analysis of whole brain proteomes data. As previously established [31, 32], it can be assumed that biological and experimental variance together weigh up to 50% of the total variance of the data; thus, any change identified in isobaric labeling beyond that threshold can be confidently considered as caused by the disease condition.

Analysis of label-free brain EV proteomes was carried out using an in-house Mascot server (version 2.6.02, Matrix Science, MA) with a precursor ion tolerance of 30 ppm and fragment ion tolerance of 0.02 Da. The UniProt human database downloaded on June 23, 2015, with 180,822 sequences and 71,773,890 residues, was used in database searching. Deamidation at N and Q and oxidation of M were set as variable modifications, while cabarmydomethyl of C was set as fixed modification. FDR < 1% was established for protein identification, and trypsin was set as proteolytic enzyme. Cluster analysis based on label-free protein quantitation (total number of spectra detected for all peptides in each protein) was performed as previously described [21] using the fuzzy c-mean algorithm [33] in R software. Only proteins with a fuzzy partition coefficient ≥ 0.5 were considered. Lists of clustered proteins were further independently analyzed by parametric one-way ANOVA with Bonferroni correction for multiple comparisons, and statistical significance was set at corrected *p* < 0.05, unless otherwise specified. Homoestadicity of data from brain EV proteomes was also analyzed by Brown-Forsythe test, and in cases where non-parametric analysis was required, one-way ANOVA on ranks was used and statistical significance was set at *p* < 0.05, unless otherwise stated. Only proteins confidently identified, based on the detailed statistical criteria, in all subjects in at least one experimental group have been reported.

A multiple correlation analysis to identify interaction between proteins in brain EV, complementary brain tissue proteome fractions, and other EV conditions was performed by calculating all potentially possible Pearson’s correlation coefficients between variables. A 95% of confidence interval was used, and strong interaction between correlated variables was considered when *r* ≥ 0.75, whereas very strong interaction between correlated variables was considered when *r* ≥ 0.85. Significance of the interaction was established when *p* < 0.05.

Analysis of MRM data from brain EV proteins was performed using Skyline software version 4.2 [30]. Parent ion identification in Skyline was achieved based on the following highly restrictive criteria: (i) peptide mass, (ii) retention time, (iii) coherent co-elution of transitions, and (iv) dot product similarity metric score dotP ≥ 0.9 [34, 35]. Thus, only proteins identified in accordance with all these criteria were considered. MRM data was reported from individual subjects.

Obtained raw data were analyzed in Microsoft Excel with the help of in-house created macros. GraphPad Prism 6 software was used for independent statistical analyses and rendering of data plots (GraphPad Software, Palo Alto, CA). All data are reported as mean ± SD, unless stated otherwise.

## Results

### Characterization of brain EV obtained from subjects with AD

**Fig. 1:**
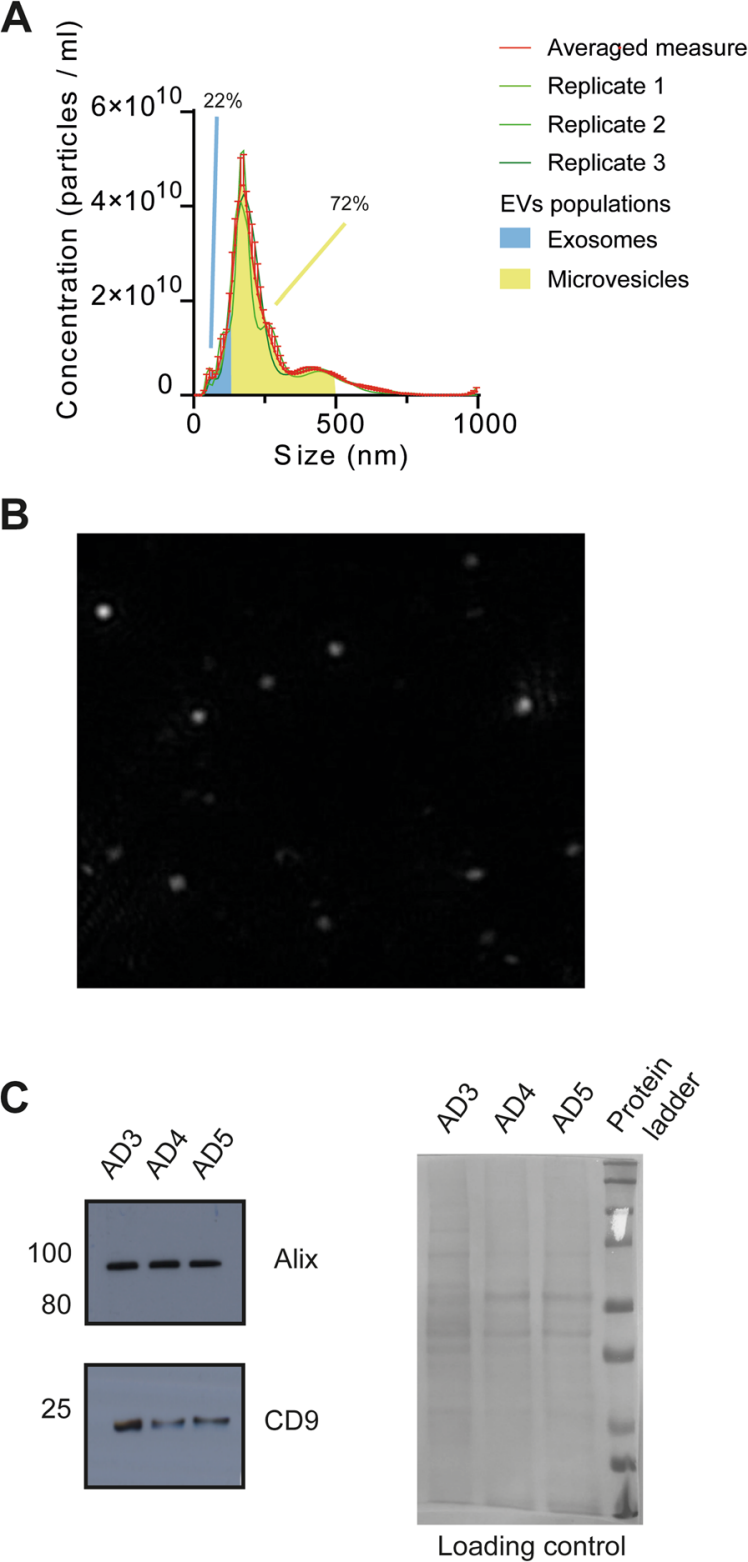
Characterization of brain-derived EV enriched by PROSPR from subjects with AD. **a** Size distribution profiles of brain EV obtained by NTA from preclinical subjects with AD. Independent runs are indicated in green, averaged distribution with standard deviation is shown in red. EV populations that qualify as exosomes by size are shaded under the curve in blue (0–150 nm) and yellow shade indicates EV populations that qualify as microvesicles (150–500 nm). **b** EV characteristic spherical shape was observed in the majority of NTA detected particles as can be seen in the representative image. **c** Presence of the exosome-specific marker Alix and the EV tetraspanin marker CD9 identified by Western blot (WB) in brain-derived EV from subjects with AD at different Braak stages (AD3–AD5). Equal protein loading is shown in the Ponceau S stained blot displayed on the right of WB results. Whole WB membranes are shown in Supplementary Figure 1

Brain vesicle content from subjects with AD obtained by PROSPR was subjected to characterization by NTA and western blot as shown in Fig. 1. NTA analyses revealed that brain vesicles in subjects with AD ranged from 15 to 995 nm in diameter. More than 20% of particles in brain EV samples qualified as exosomes by size (diameter < 159 nm) and shape (spherical) (Fig. 1a, b). Similarly, > 70% of the total vesicles identified qualified as lower-range spherical microvesicles (diameter < 500 nm), as shown in Fig. 1a. Western blot data confirmed the presence of the specific EV markers Alix and CD9 in the brain EV preparations obtained, as shown in Fig. 1c and Supplementary Figure 1. Finally, although an apparent decrease in the presence of the EV-specific marker tetraspanin CD9 was observed along the analyzed Braak stages by western blot (Fig. 1c) and LC-MS/MS (not shown), this decrease did not reach statistical significance. Additionally, ultrastructural characterization by cryo-electron microscopy of circulating EV isolated by PROSPR, as performed previously [25], confirmed the predominant presence of spherical vesicles in the EV preparations obtained by this method.

### AD progression modulates EV biogenesis in the affected brains

**Fig. 2:**
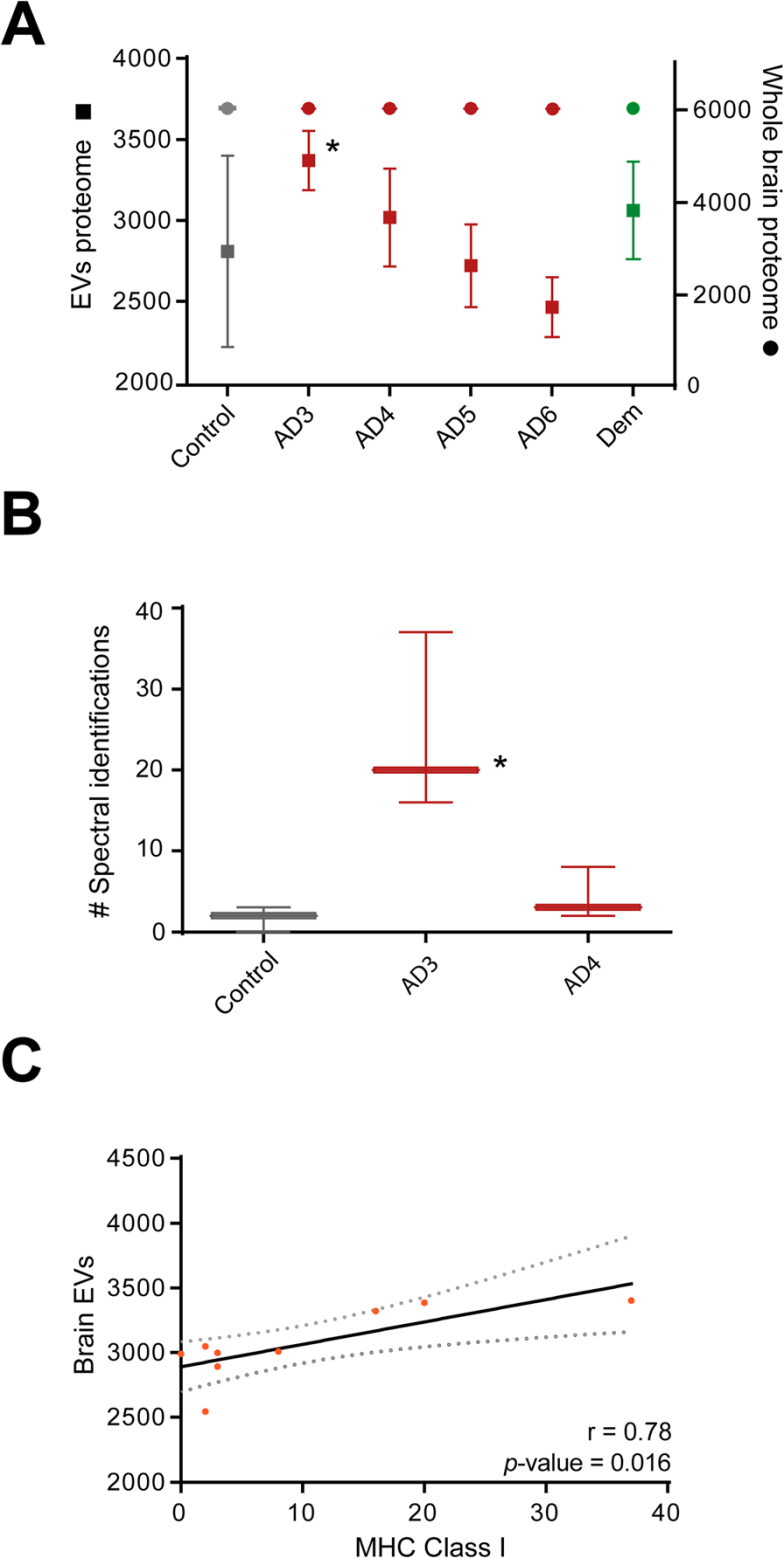
Analysis of brain EV biogenesis in AD. **a** Analysis of the total number of proteins identified from brain EV proteomes and remaining whole brain proteomes throughout the progressive course of AD (AD3 to AD6) in front of age-matched controls (control) and mixed dementias (Dem) subjects. **b** Relative quantitation of MHC class I markers proteins in brain EV. Significant differences were only observed for this family of protein in preclinical AD3 versus age-matched controls. Conditions AD5 and AD6 were not displayed as these did not shown significant differences with age-matched controls. Significant differences versus age-matched controls were analyzed by one-way ANOVA with Bonferroni correction for multiple comparisons. *Significant differences compared to age-matched controls *P* < 0.05. **Significant differences compared to age-matched controls *P* < 0.01. **c** Correlation between brain EV proteomes and MHC class-derived proteins identified in preclinical AD

We sought to investigate any change in brain proteomes and corresponding EV proteomes that could relate to alterations in EV biogenesis over the progression of AD. As shown in Fig. 2a, while no significant changes were observed in the total number of proteins in brain tissue proteomes, the total number of proteins in brain EV was significantly boosted during the preclinical stage of the disease. Additionally, a gradual downward tendency in the number of global EV proteomes over the course of AD could be perceived, although it was only apparent (Fig. 2a). Likewise, a slight increase in the number of global EV proteomes in the brains of subjects affected by mixed dementias was also found, although it did not reach statistical significance (Fig. 2a).

Subsequently, to investigate whether the observed significant modulation in the number of global EV proteomes observed in preclinical AD was due to an increase in protein cargo or, on the contrary, was related to a modulation of EV biogenesis in this disease stage, we investigated the presence and abundance of specific EV markers in these EV data. For these analyses, we mined data from the top 50 EV markers, as described in our previous work [14], curated in specialized EV databases (i.e., Exocarta and Vesiclepedia). We found no significant differences in any of the top 50 EV markers investigated, except for the specific family of markers MHC class I, which was found to be significantly upregulated in EV from preclinical AD, as shown in Fig. 2b. Therefore, to confirm a potential link between the increase in MHC class I marker-containing EV populations and the increase in the number of EV proteins observed in preclinical AD, we performed a correlation study. A positive and significant interaction between these variables was identified (*r* = 0.78 and *p* value = 0.016) (Fig. 2c), reinforcing the notion that EV biogenesis in preclinical AD becomes augmented due to an increment in a specific subpopulation of EV characterized by MHC class I markers.

### Clustering analysis of brain EV proteomes in AD reveals archetypal molecular patterns of disease progression

**Fig. 3:**
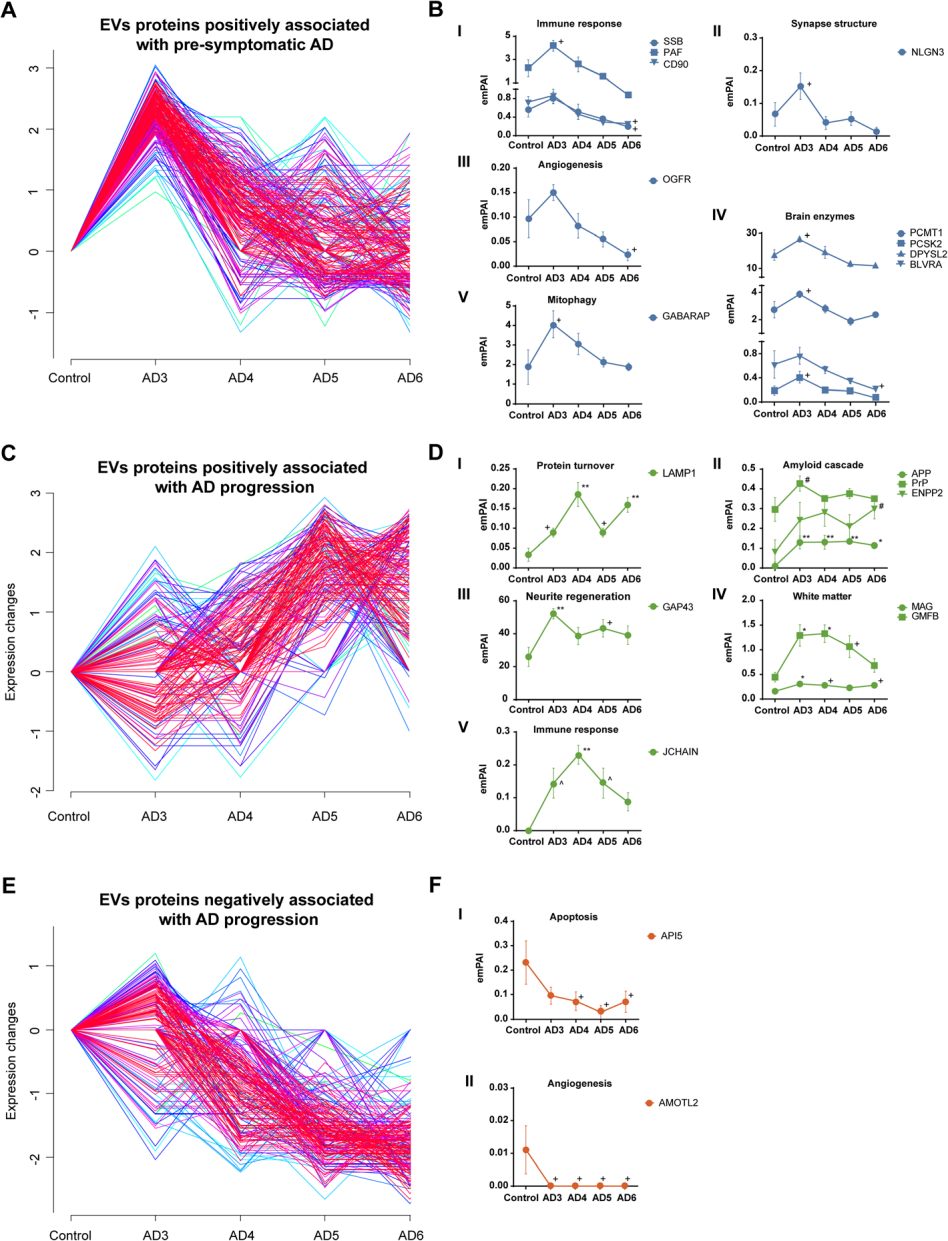
Temporal clustering analysis of brain EV proteomes in AD. Fuzzy c-mean clustering of brain EV proteome was performed based on total number of spectra identified for each protein by label-free quantitative proteomics. Age-matched controls (control) were used as baseline in clustering analyses. Negative expression changes refer to lower concentration in AD compared to baseline. Proteins with low deviation were indicated by warm colored traces (red/magenta), while proteins with high deviation were colored in cold colors (blue/green/cyan). A total of 10 protein clusters were obtained in these analyses. **a**, **c**, **e** Cluster 8, cluster 7, and cluster 9, respectively (remaining clusters are displayed in Supplementary Figure 1 in the online-only Supplementary Material). Clustered proteins were further analyzed by one-way ANOVA with Bonferroni correction for multiple comparisons, unless otherwise specified. Significant differences are expressed versus controls. **b**, **d**, **f** Quantification of proteins significantly modulated from clusters 8, 7, and 9, respectively. *Significant differences *P* < 0.05. **Significant differences *P* < 0.01. ^+^Significant differences determined by ANOVA with Fisher’s LSD *P* < 0.05. ^#^Significant differences determined by nonparametric one-way ANOVA on ranks *P* < 0.05. ^Significant differences determined by nonparametric one-way ANOVA with Holm-Šídák correction *P* < 0.05

Characterization of EV enriched from brain tissues by shotgun proteomics led to the identification of a total of 9631 proteins from all analyzed conditions (Supplementary Data Set 1). To obtain a visual representation of this complex and large dataset and to categorize identified brain EV proteins according to their abundances and distribution along the AD course, we performed fuzzy c-mean clustering as previously described [2, 3]. A total of 10 clusters containing 332 proteins on average were obtained, from which three showed distinctive and remarkable patterns (Supplementary Figure 2 and Supplementary Data Set 2). Cluster 8 specifically contained those proteins positively associated with presymptomatic AD, with a protein abundance distribution that clearly peaked in the AD3 condition and gradually decreased in abundance from AD4 onwards (Fig. 3a). Clustered proteins positively associated with preclinical AD were further individually analyzed, revealing a significant boost of the immune response in presymptomatic AD (modulated SSB, PAF, and Thy-1 membrane glycoprotein CD90 proteins) and modulation of the synapse structure (NLGN3) and the angiogenesis-associated protein OGFR (Fig. 3b—I to III). Furthermore, an important subset of brain enzymes, including PCMT1, PCSK2, DPYSL2, and BLVRA, displayed a positive association with presymptomatic AD, as shown in Fig. 3b—IV, and significant dysregulation of the mitophagy and phagophore-associated protein GABARAP was also observed in this AD3-positively associated cluster (Fig. 3b—V).

In addition, proteins in cluster 7 displayed a positive association with the progressive course of AD (Fig. 3c). In-depth analysis of these clustered proteins revealed altered lysosome dynamics during disease progression, with significantly dysregulated lysosome-associated membrane glycoprotein 1 (LAMP1) (Fig. 3d—I). As expected, multiple amyloid cascade-associated proteins were included in this cluster as significantly modulated in brain EV by the course of illness (i.e., APP, PrP, and ENPP2) (Fig. 3d—II). Similarly, the presence in brain EV of the neurite regeneration-associated protein GAP43 (Fig. 3d—III) and white matter-related proteins MAG and GMFB (Fig. 3d—IV) was also positively associated with AD progression. Interestingly, immunoglobulin J chain (JCHAIN) was also included in cluster 7, which demonstrates a potential shift in immune response-associated proteins in EV from preclinical to symptomatic AD stages (Fig. 3d—V). In contrast, there was a large subset of proteins collected in cluster 9 displaying a negative association with AD progression (Fig. 3e). As shown in Fig. 3f, further analysis of these clustered molecules revealed a gradual and significant decrease in the abundance of the apoptosis-associated protein API5 in EV (Fig. 3f—I). Of note, we observed a total absence of the angiogenesis-related protein AMOTL2 in all AD stages compared to that in the control (Fig. 3f—II). Finally, from the analysis of all populations of EV proteins detected in AD conditions by fuzzy c-means, it is worth mentioning that common patterns in EV composition between AD and mixed dementias could only be identified in the preclinical stage of AD (see graphic of cluster 2 in Supplementary Figure 3). This reinforces the importance of the vascular component in the preclinical phases of the disease and its impact on the compositions of these vesicles, as we recently found [4].

### Parallel analysis of brain EV compositions and whole brain quantitative profiles

Proteins identified as significantly modulated in brain EV proteomes (as shown in Fig. 3b, d, f) were further analyzed and compared in parallel to their levels in tag-quantified remaining brain proteomes from subjects with AD (Supplementary Data Set 3). For these analyses, we performed a correlation study, and the obtained data are shown in Table 1. Of note, we observed a very strong positive association (*r* = 1) between the levels of white matter-associated protein MAG in the remaining brain fractions of AD5 subjects and the levels of the protein in brain EV from AD6 subjects, indicating potential degradation of this protein through EV in the advanced-to-terminal stages of the disease. On the other hand, based on the tendency observed for the glial protein GMFB in brain EV of subjects with AD (see Fig. 3d—IV) and the positive correlations obtained for this protein between remaining brain AD3 levels versus levels in brain EV from subjects with AD4 and AD6, we can assume that encapsulation of GMFB in EV represents a preclinical potentially neuroprotective strategy that gradually decays over the clinical course of AD (Table 1). Similarly, the phagophore enclosing protein GABARAP was high in the presence of brain EV during the preclinical stage of AD, followed by a gradual decrease in abundance throughout AD progression. This distribution, together with the set of positive correlations detected for this phagophore-associated protein between EV and whole brain abundances, strikingly indicates disrupted mitophagy during the clinical course of AD. Thus, we also found that the levels of this protein in the temporal lobe of subjects with AD went from stable to apparent downregulation over the progression of the disease.

**Table 1:** Statistically significant correlations detected for individual proteins quantified at different disease Braak stages in brain extracellular vesicles (EVs) and remaining brain tissues

Protein gene symbol	Correlated variables		Correlation coefficient*	***p*** value	Potential interpretation**
MAG	AD5 WB	AD6 EVs	0.99	0.040	Degradation by EVs
GMFB	AD3 WB	AD4 EVs	0.99	0.048	Preclinical and early symptomatic rescue response in EVs
	AD3 WB	AD6 EVs	0.99	0.049	–
GABARAPL	AD3 WB	AD3 EVs	0.99	0.041	–
	AD3 EVs	AD4 WB	1.00	0.003	Preclinical encapsulation in EVs
	AD3 EVs	AD5 WB	1.00	0.013	Preclinical encapsulation in EVs
	AD3 EVs	AD6 EVs	0.86	0.003	Abnormal uptaking late stage AD
Lamp1	AD3 EVs	AD4 WB	0.99	0.039	Ongoing Lamp1 synthesis during AD progression. Abnormal Lamp1 inclusion in lysosomes in AD progression
	AD3 EVs	AD5 WB	1.00	0.020	
	AD3 EVs	AD6 WB	0.99	0.036	
GAP43	AD3 WB	AD4 EVs	0.99	0.046	Preclinical and early symptomatic rescue response in EVs

Correlations were calculated based on abundance of proteins identified in brain EVs and remaining brain tissues

*Refers to Pearson correlation coefficient

**Plausible interpretation of the protein role(s) based on the correlated variables involved in each identified statistically significant interaction(s) (parallel analysis brain EVs versus remaining brain tissues)

From this correlation study, we also obtained striking data about the lysosome and autophagy-associated protein LAMP1. We found that although synthesis of LAMP1 in the remaining whole brain was upregulated in AD stages (Supplementary Figure 4), inclusion of LAMP1 into lysosomes was disrupted from the preclinical stage of the disease, as shown in Table 1. The neurite regeneration protein GAP43 instead showed a positive and significant progressive correlation between the whole brain in AD3 and EV in AD4 (Table 1).

### Validation of proteins in brain EV by multiple reaction monitoring

**Fig. 4:**
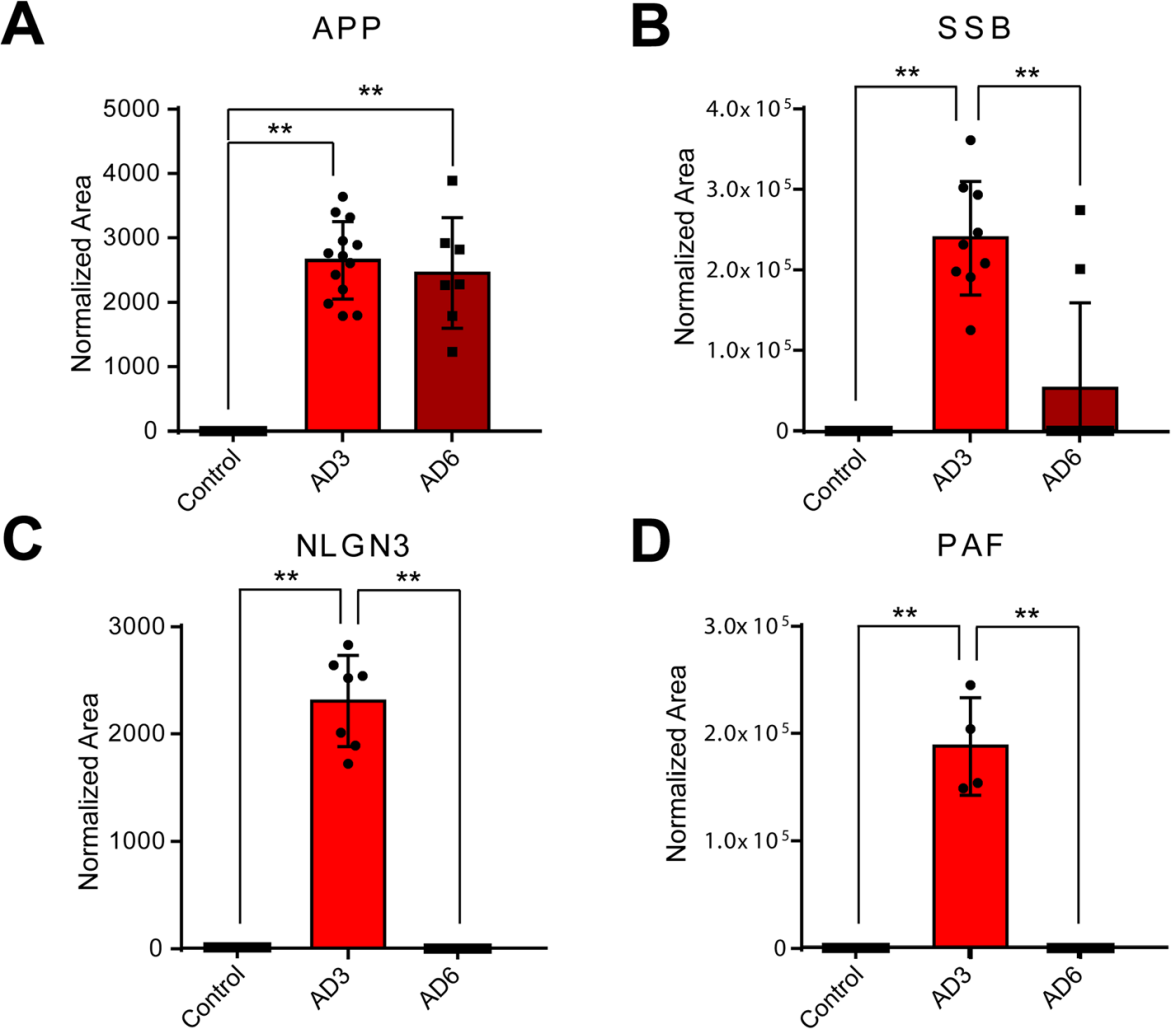
Multiple reaction monitoring (MRM) analysis of brain EV proteins. Proteins identified in brain EV in subjects with AD during the discovery phase were further analyzed by MRM. MRM-based relative quantitation of **a** amyloid precursor protein (APP), **b** single-stranded binding protein (SSB), **c** neuroligin-3 (NLGN3), and **d** platelet-activating factor (PAF). Differences were analyzed by one-way ANOVA with Bonferroni correction for multiple comparisons. *Significant differences *P* < 0.05. **Significant differences *P* < 0.01

To validate the presence and level of relevant proteins identified in brain EV in this study, we used an MRM proteomics strategy as previously reported [36–38]. Identification and quantification of proteins by MRM was based on the average of 4–6 transitions for every precursor peptide. Using this restrictive analytical approach, four proteins (APP, NLGN3, PAF, and SSB) were confidently identified and quantified in this study (Fig. 4). The amyloid precursor protein APP was present at very similar levels in preclinical and advanced clinical AD stages, with no significant differences observed between conditions (Fig. 4a). The DNA-binding protein SSB was significantly upregulated during the preclinical stage of AD in brain EV (Fig. 4b). In addition, neuroligin NLGN3 and PAF were significantly upregulated in preclinical AD, and their presence in AD6 did not reach the level of detection (Fig. 4c, d). This was expected for some proteins and conditions considering the lower sensitivity of the triple quadrupole mass spectrometer compared to that of a high-throughput Orbitrap mass spectrometer [39]. None of these validated proteins in brain EV from subjects with AD could be found at detectable levels in brain EV from age-matched controls.

Validation of EV proteins at the peptide level, as performed here, is somewhat limited due to the characteristic low abundance of protein cargoes/receptors in these vesicles. Nevertheless, in this study, we were able to validate the identification and relative abundance of several brain EV proteins in individual subjects, a fact that reinforces the substantiality and suitability of these vesicles in innovative diagnostic approaches to AD.

## Discussion

Brain EV holds promise to assist in the treatment of AD-type neurodegeneration and its associated cognitive signs [40, 41]. This intercellular communication mechanism has been implicated in the spreading of neurodegenerative culprits from cell-to-cell in the brain, and suppression of EV biogenesis has been recently hypothesized as a viable strategy for slowing AD neurodegeneration [6]. However, to advance the achievement of novel therapeutic applications and to better understand the contributions of these vesicles to AD neuropathology, their divergent compositions throughout the progressive course of AD and potential alterations in their biogenesis need to be uncovered, as recently noted [42]. Enrichment of EVs from brain tissues can be considered a challenging task and the presence of contaminants from brain tissues in the obtained EV fractions cannot be overlooked. Thus, the methodology employed here previously shown that whole organelle contamination in brain EV preparations can be kept below 10% (particles larger than 700 nm in diameter) [14], a result consistent with the NTA data obtained in this study. However, we still consider that whole brain contamination in brain EV preparation is a challenging limitation that requires of further research, by complementary methodologies, and of continuous improvement.

In this study, our systems biology approach revealed that EV biogenesis becomes altered during the preclinical stage of AD linked to an increase in the genesis of a specific population of EV that express the MHC class-type markers. These findings are partially consistent with previous reports in other brain diseases [15]. EV containing MHC class antigens have been vastly attributed to bone marrow dendritic cells (DCs) and brain microglia cells [43, 44]. Several studies indicate that DCs alter their functional properties and release proinflammatory molecules in the presence of amyloid-β toxicity [45, 46]. Based on the findings reported here, the interaction between DCs and amyloid-β is mediated by EV. Additionally, these data do not support the hypothesis that an increase in brain EV biogenesis would be caused by interactions of the Aβ peptide and hyperphosphorylated Tau with the endocytic pathway, as previously hypothesized [6]. An increase in EV biogenesis was only found to be significantly evident in preclinical AD in this study; thus, our findings align better with previous reports that show mild-to-moderate hypoxia as the core cause of the boost in EV biogenesis in the diseased brain [47]. This is especially manifested when we consider that brain EV manifest the influence of the AD vascular component during the preclinical stage of the disease [17]. Similarly, we found by advanced proteome clustering that common patterns between mixed dementias and all analyzed AD stages could only be identified in brain EV from preclinical AD, a finding that reaffirms our recently suggested hypothesis indicating that the influence of the vascular component in brain EV may decay over the course of AD [17]. Although we found that the amyloid cascade seems to not interfere with EV biogenesis, especially throughout the disease course, an abrupt increase in the levels of amyloid precursor protein APP in brain EV during preclinical AD was encountered. We also observed that this increase in APP in brain EV was maintained throughout the whole AD neuropathology. The presence of APP in brain EV from experimental AD models was initially reported in 2012 by Perez-Gonzalez [48], although very recently, it has also been shown that APP is present in brain EV from subjects with AD at higher levels compared to age-matched controls, as we have also observed here. These authors also found that these EV loaded with APP show the ability to spread Aβ toxicity cell-to-cell [49]. Similarly, previous in vitro and in vivo studies have linked the upregulated presence of APP in EV to the upregulated presence of the prion protein PrP in these vesicles [49]. However, this co-upregulation was not confirmed in clinical subjects with AD until here, when we found significant co-upregulation of the prion protein PrP and APP in brain EV from subjects with AD compared to age-matched controls. According to these data, PrP was at its highest level in brain EV during the preclinical stage of the disease. The interaction between APP and PrP in brain cells has been largely described [50, 51], although the purpose of that interaction in brain EV remains unknown [42]. The available data about the interactions between these proteins come mainly from Falker and colleagues, who proposed that PrP in EV may contribute to sequestering toxic amyloid oligomers [52]. Our findings throughout AD progression support this hypothesis; hence, a significant increase in PrP in brain EV was uniquely manifested during the preclinical stage of AD, and the presence of PrP in brain EV was reduced to similar levels as in age-matched controls at the initial manifestation of clinical signs. Thus, based on our data, we consider that PrP may work as a switch activating the sequestration of APP in brain EV, a mechanism that abruptly decays in the temporal brain after passing through the preclinical stage of the disease. Furthermore, our results do not support the hypothesis that brain EV enriched with toxic amyloid oligomers and PrP contribute to increased levels of aggregates in AD brains; hence, co-upregulation of both proteins in brain EV is highly active only during the preclinical stage of the disease. In line with these findings, an extensive review analysis of the CNS-derived EV literature in the context of AD is included in Table 2 and indicates that circulating neuronal EV contain Aβ1-42 up to 10 years prior to the clinical onset of AD [53]. Similarly, several studies demonstrate that circulating EV exert potential diagnostic/prognostic abilities in AD dementias [54–58]. Furthermore, the upregulated presence of phosphorylated Tau residues in circulating brain EV from cerebrospinal fluid was encountered in preclinical subjects with AD (Braak stage 3) [59]. We also found that brain EV are significantly loaded with higher levels of essential brain enzymes during the preclinical stage of the disease, which included protein-l-isoaspartate (d-aspartate) o-methyltransferase (PCMT1), neuroendocrine convertase 2 (PCSK2), dihydropyrimidinase-like 2 (DPYLS2), and biliverdin reductase-A (BLVRA). All these enzymes have previously been implicated in AD [60–63]. We also observed that the levels of these enzymes in brain EV, albeit upregulated in preclinical AD, were significantly downregulated throughout the entire disease progression. Similarly, we found that the levels of the immunoglobulin J chain in brain EV gradually and significantly increased during the preclinical and early symptomatic stages of AD, whereas these levels were downregulated in the terminal stages of the disease. The observed increase in immunoglobulin J chain can be associated with enhanced permeability of the blood brain barrier and an increased supply of outer EV to the diseased CNS [64].

**Table 2:** Extensive review analysis of CNS-derived EVs findings in the context of AD

Exosome-associated protein	Protein modulation	EV source	Analytical technique	Observations	Reference
P-S396-tau, P-T181-tau and Aβ1-42	Upregulation in AD	NDE	Immunoassay	Predictor of AD development prior to clinical onset	[54]
SNAP-25	Downregulation in AD	NDE	Immunoassay	Negative correlation between the levels of SNAP-25 and cognitive status	[55]
Gelsolin	Downregulation in DLB compared to AD	Plasma-derived EVs	Proteomics	Potential biomarker able to differentiate DLB and AD	[56]
Growth-associated protein 43 and synapsin 1	Downregulation in AD but not in dementia patients	NDE	Immunoassay	Possible early differential diagnosis marker to differentiate AD and dementia	[57]
β/γ-secretase and sAPPβ	Upregulation in AD	NDE	Immunoassay	Astrocyte-derived exosomes of AD patients show up to 20-fold upregulation than neuron-derived exosomes	[58]
pS396 tau and Aβ	Upregulation in AD	Cortical grey matter EVs	Immunoassay	–	[59]
ANXA5, VGF, GPM6A and ACTZ	Presence	Cortical grey matter EVs	Quantitative proteomics and machine learning	EVs signature panel of proteins in AD	[59]
Total and phosphorylated tau	Upregulation in AD	CSF from AD Braak stage 3	Immunoassay	Considered patients with mild AD	[60]

*NDE* plasma circulating neuronal-derived exosomes, *DLB* dementia with Lewy bodies, *CSF* cerebrospinal fluid

In-depth analysis of brain EV proteomes in parallel to the remaining brain proteomes, as performed here, also demonstrated that brain EV serve to encapsulate/degrade proteins during disease progression. Unexpectedly, we found that the myelin protein MAG serves as a clear example of this functionality, a fact that probably contributes to the progression of white matter disease in AD and its associated cognitive signs, as previously speculated [65]. In contrast, considering the protective nature of the glial maturation factor GMFB, we observed that encapsulation of this protein in EV during the preclinical stage of AD potentially serves neuroprotective aims. Similarly, the neurite regeneration protein GAP43 was also found in similar patterns potentially contributing to neuroprotective purposes in AD, although these mechanisms were found to be disrupted from advanced to terminal AD stages.

Strikingly, we also observed that inclusion of GABARAP in phagophore vesicles was disrupted after passing through the preclinical stage of AD, coinciding with the manifestation of clinical symptoms. GABARAP becomes essential in the process of engulfing damaged mitochondria into phagophores and during the proper closure of active vesicles in mitophagy [66]. This mechanism, based on our data, was only functional during preclinical AD. Similarly, disrupted autophagic degradative flow was indicated here by evident failure to include LAMP1 in EV (lysosomes), although the levels of the protein were significantly upregulated in brain tissues during the whole AD course.

## Conclusions

In conclusion, these novel and important data collectively indicate that although lysosome and phagophore formation is highly active in brain cells during the preclinical stage of AD, these cells fail to contribute to proper and progressive autophagic flow throughout AD neuropathology; thus, a large number of these degradative vesicles enter the EV pathway from the point of clinical AD (Braak 4). We also show that these vesicles contain damaged mitochondrial organelles associated with an increase in GABARAP in preclinical AD, as well as contain the prion proteins PrP and APP. Thus, our data undoubtedly support incipient evidence indicating that impaired autophagy in the temporal lobe contributes to the spreading of AD neuropathology through EV [67]. Furthermore, we show for the first time that preclinical AD is the crucial disease stage in which autophagy-related mechanisms take potential pro-neurodegenerative pathways.

## Supplementary information

**Additional file 1.**

**Additional file 2.**

**Additional file 3.**

**Additional file 4.**

## Data Availability

As recently pointed by its relevance for the neuroscientific community [68], all data generated in this study were made publicly available via the ProteomeXchange consortium in the partner repository PRIDE. Identifier PXD015578. Additionally, whole membranes of western blot experiments were included as supplementary material.

## Abbreviations

Aβ peptide: Fibrillar amyloid-β
AA: Ammonium acetate
ABB: Ammonium
ACN: Acetonitrile
AD: Alzheimer’s disease
CNS: Central nervous system
DCs: Dendritic cells
DTT: dithiothreitol
EV: Extracellular vesicles
FA: Formic acid
FDR: False discovery rate
FT-MS: Full Fourier transform mass spectrometry
HPLC: High-pressure liquid chromatography
IAA: Iodoacetamide
LC-MRM-MS: Liquid chromatography multiple reaction monitoring mass spectrometry
MRM: Multiple reaction monitoring
SDC: Sodium deoxycholate
TEAB: Triethylammonium bicarbonate
