# Supplementary material for: Alzheimer’s disease progression characterized by alterations in the molecular profiles and biogenesis of brain extracellular vesicles
Source: Alzheimers Res Ther. 2020 May 8;12:54. doi: 10.1186/s13195-020-00623-4 (PMC7210691; doi:10.1186/s13195-020-00623-4)
Supplement: Supplementary file 1 — Additional file 1. [file 13195_2020_623_MOESM1_ESM.pdf]

SUPPLEMENTARY MATERIAL

Alzheimer's disease progression characterized by alterations in the molecular profiles and biogenesis of brain extracellular vesicles

Xavier Gallart-Palau [1,2, §], Xue Guo [2, §], Aida Serra [1,*], Siu Kwan Sze [2,*]

[1] IMDEA-Food Research Institute, +Pec Proteomics, Campus of International Excellence UAM+CSIC, Old Cantoblanco Hospital, 8 Crta. Canto Blanco, Madrid, 28049, Spain.

[2] School of Biological Sciences, Nanyang Technological University, 60 Nanyang Drive, Singapore 637551.

[§] These authors contributed equally.

* Corresponding authors (Siu Kwan Sze, Email: sksze@ntu.edu.sg; Aida Serra, Email: aida.serra@imdea.org).

Table of content:

**Content**	**Page**
Supplementary Table 1: Detail of the human post-mortem brains analyzed in this study.	S-2
Supplementary Table 2: List of precursor ions and transitions used for protein validation by MRM.	S-3
Supplementary Figure 1: Western blot whole membranes.	S-4
Supplementary Figure 2: Fuzzy c-mean clustering of proteomic data. Graphic for protein clusters 1, 3-6 and 10.	S-5
Supplementary Figure 3: Brain EVs commonalities in preclinical AD and mixed dementias.	S-6
Supplementary Figure 4: Fold-change ratio of LAMP1 in whole brain over the AD progression.	S-7
Supplementary Data Set 1: List of proteins identified in brain EVs from age-matched controls (Control), AD patients at different braak stages (AD3 to AD6) and Mixed Dementia (Dem).	Separate file
Supplementary Data Set 2: List of proteins clustered in the 10 clusters obtained from the Fuzzy c-mean clustering analysis.	Separate file
Supplementary Data Set 3: List of proteins identified from whole brain of Alzheimer´s patients (braak stages 3- 6, as AD3 to AD6) and age-matched controls.	Separate file

Supplementary Table 1. Detail of the human post-mortem brains analyzed in this study. [a]PMD refers to post-mortem delay expressed in hours.

	Age	Gender	PMD[a]
**Controls**			
1	69	M	18.4
2	72	M	24.9
3	67	M	23.5
**AD3**			
1	72	M	27.3
2	78	M	23.3
3	73	M	21.2
**AD4**			
1	81	F	12.9
2	91	F	12.4
3	88	F	20.7
**AD5**			
1	63	M	17.5
2	76	M	11.1
3	68	M	21.2
**AD6**			
1	73	M	15.4
2	68	M	6.2
3	63	M	16.9
**VaD**			
1	74	M	13.6
2	65	M	7.8
3	63	M	30.5

Supplementary Table 2. List of precursor ions and transitions used for protein validation by MRM.

Protein	Peptide selected	Precursors m/z **	Product m/z***
APP	LVFFAEDVGSNK	663.3404 ++	F [y10] - 1113.5211+
			F [y9] - 966.4527+
			A [y8] - 819.3843+
			D [y6] - 619.3046+
			V [y5] - 504.2776+
			G [y4] - 405.2092+
NLGN3	ELVEQDIQPAR	649.3410 ++	V [y9] - 1055.5480+
			E [y8] - 956.4796+
			Q [y7] - 827.4370+
			D [y6] - 699.3784+
			I [y5] - 584.3515+
			P [y3] - 343.2088+
PAF	TAPYVVTGSVDQTVK	782.9145 ++	V [y11] - 1132.6208+
			V [y10] - 1033.5524+
			T [y9] - 934.4840+
			G [y8] - 833.4363+
			S [y7] - 776.4149+
			D [y5] - 590.3144+
			Q [y4] - 475.2875+
			P [b3] - 135.5761++
SSB	FASDDEHDEHDE**N**GATGPVKR*	582.5006 ++++	N [y9] - 900.4898+
			G [y8] - 785.4628+
			T [y6] - 657.4042+
			G [y5] - 556.3566+
			P [y4] - 499.3351+

* Refers to a peptide containing a missed cleavage site.
** State designated with + symbols.
***Initial transition ion residue [ion designation] m/z and charge state designated with + symbols.

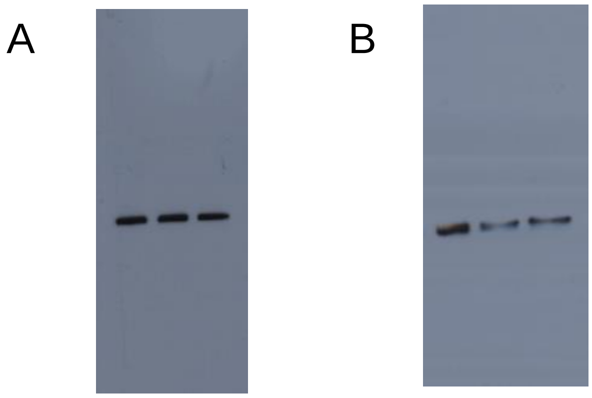

Supplementary Figure 1: Western blot whole membrane for **A.** detection of Alix and **B.** detection of CD9 in brain-derived EVs from AD patients (Braak stage 3).

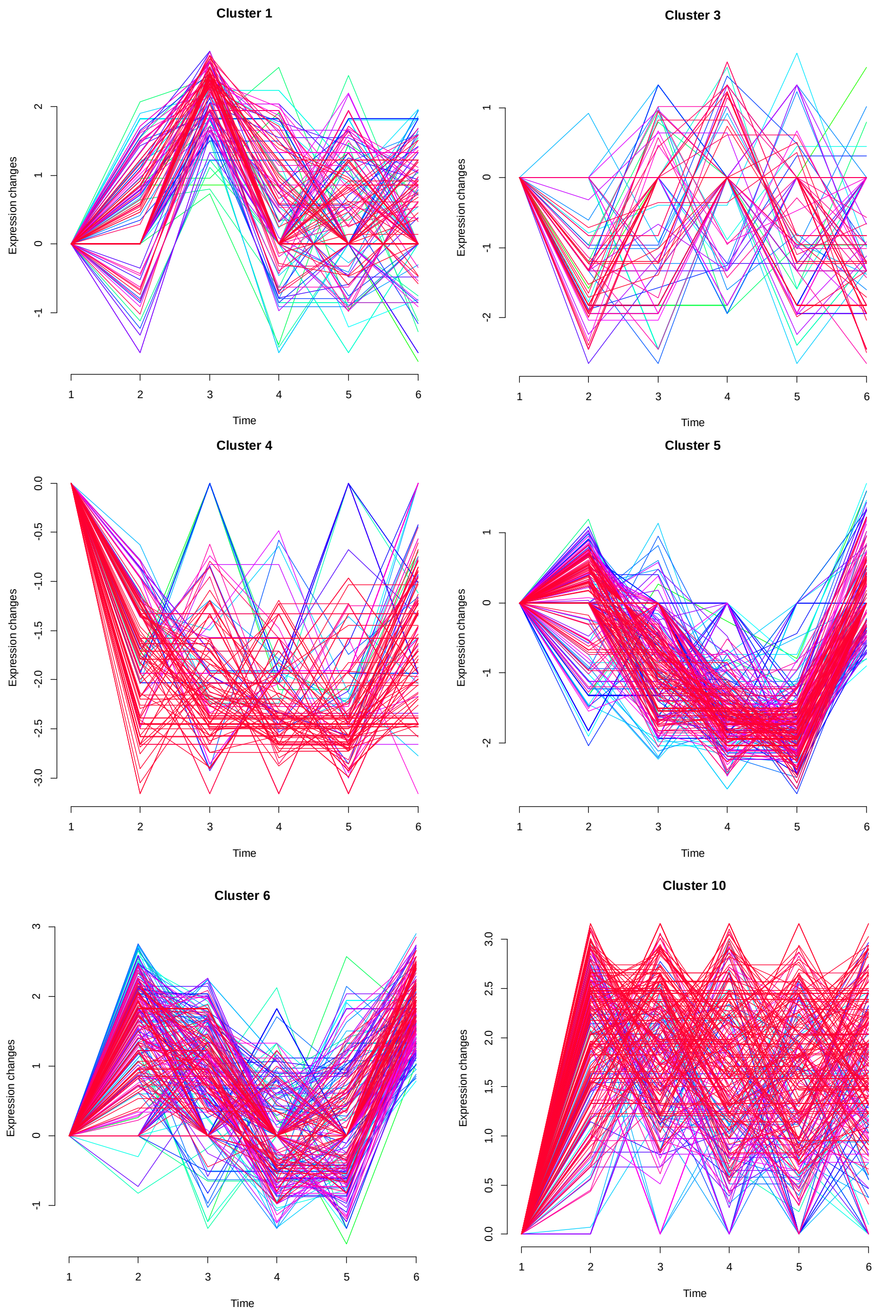

Supplementary Figure 2: Fuzzy c-mean clustering of proteomic data. Graphic for protein clusters 1, 3-6 and 10. The protein abundance used for the clustering analysis was expressed as total number of spectra identified for every protein by label-free quantitative proteomics. Control was used as baseline. In the graphics warm colored lines correspond to proteins with higher deviation in the cluster while cold colored lines correspond to proteins with lower deviation.

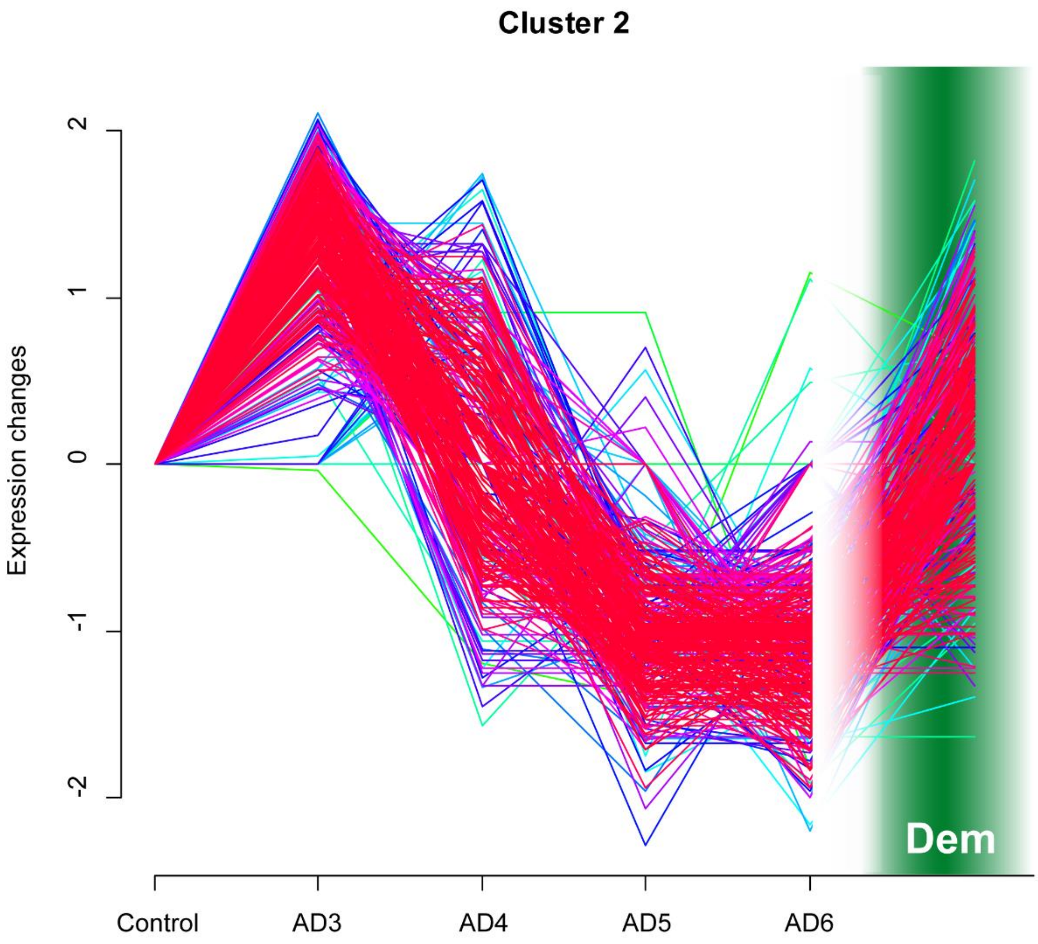

Supplementary Figure 3. Brain EVs commonalities in preclinical AD and mixed dementias. Proteins in preclinical AD clustered in Cluster 2 displayed common levels in brain EVs from AD3 and mixed dementias (Dem) subjects. Dem was shaded in green.

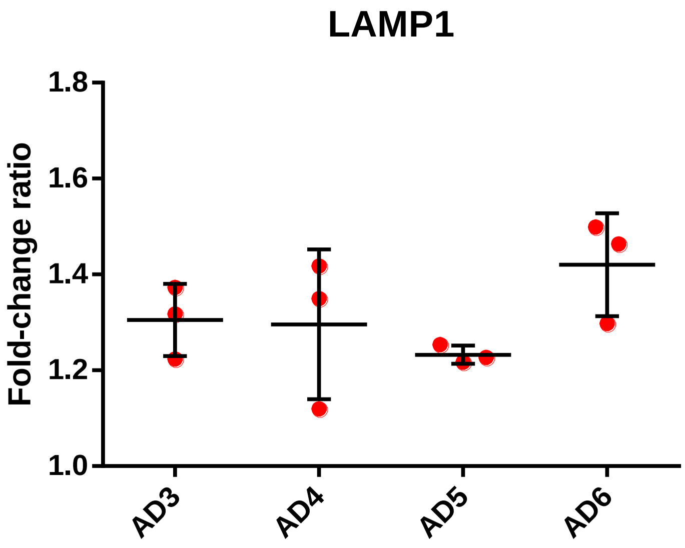

Supplementary Figure 4. Fold-change ratio of LAMP1 in whole brain over the AD progression. Control is used as reference to calculate the fold-change ratio for every condition.
